# Supplementary material for: Resolving the full spectrum of human genome variation using Linked-Reads
Source: Genome Res. 2019 Apr;29(4):635–45. doi: 10.1101/gr.234443.118 (PMC6442396; doi:10.1101/gr.234443.118)
Supplement: Supplemental Material [file supp_gr.234443.118_Supplemental_longranger-2.2.zip › longranger-2.2.tar.bz2!/lr2.2-public/lib/stan/cmdstan/stan/doc/api/html/gamma_8hpp.html]

Stan: src/stan/prob/distributions/univariate/continuous/gamma.hpp File Reference


|  |  |
| --- | --- |
|  | Stan  2.5.0  probability, sampling & optimization |


- Main Page
- Related Pages
- Namespaces
- Classes
- Files

- File List
- File Members

All Classes Namespaces Files Functions Variables Typedefs Enumerations Enumerator Friends Macros Pages

- src
- stan
- prob
- distributions
- univariate
- continuous

Namespaces |
Functions

gamma.hpp File Reference

`#include <boost/random/gamma_distribution.hpp>`  
`#include <boost/random/variate_generator.hpp>`  
`#include <stan/agrad/partials_vari.hpp>`  
`#include <stan/math/error_handling.hpp>`  
`#include <stan/math/constants.hpp>`  
`#include <stan/math/functions/multiply_log.hpp>`  
`#include <stan/math/functions/value_of.hpp>`  
`#include <stan/meta/traits.hpp>`  
`#include <stan/prob/constants.hpp>`  
`#include <stan/prob/traits.hpp>`  
`#include <stan/prob/internal_math.hpp>`

Go to the source code of this file.

|  |  |
| --- | --- |
| Namespaces | |
|  | stan |
|  | Probability, optimization and sampling library. |
|  | |
|  | stan::prob |
|  | Templated probability distributions. |
|  | |

|  |  |
| --- | --- |
| Functions | |
| template<bool propto, typename T\_y , typename T\_shape , typename T\_inv\_scale > | |
| return\_type< T\_y, T\_shape,   T\_inv\_scale >::type | stan::prob::gamma\_log (const T\_y &y, const T\_shape &alpha, const T\_inv\_scale &beta) |
|  | The log of a gamma density for y with the specified shape and inverse scale parameters. More... |
|  | |
| template<typename T\_y , typename T\_shape , typename T\_inv\_scale > | |
| return\_type< T\_y, T\_shape,   T\_inv\_scale >::type | stan::prob::gamma\_log (const T\_y &y, const T\_shape &alpha, const T\_inv\_scale &beta) |
|  | |
| template<typename T\_y , typename T\_shape , typename T\_inv\_scale > | |
| return\_type< T\_y, T\_shape,   T\_inv\_scale >::type | stan::prob::gamma\_cdf (const T\_y &y, const T\_shape &alpha, const T\_inv\_scale &beta) |
|  | The cumulative density function for a gamma distribution for y with the specified shape and inverse scale parameters. More... |
|  | |
| template<typename T\_y , typename T\_shape , typename T\_inv\_scale > | |
| return\_type< T\_y, T\_shape,   T\_inv\_scale >::type | stan::prob::gamma\_cdf\_log (const T\_y &y, const T\_shape &alpha, const T\_inv\_scale &beta) |
|  | |
| template<typename T\_y , typename T\_shape , typename T\_inv\_scale > | |
| return\_type< T\_y, T\_shape,   T\_inv\_scale >::type | stan::prob::gamma\_ccdf\_log (const T\_y &y, const T\_shape &alpha, const T\_inv\_scale &beta) |
|  | |
| template<class RNG > | |
| double | stan::prob::gamma\_rng (const double alpha, const double beta, RNG &rng) |
|  | |


---

[ Stan Home Page ]

*© 2011–2014,
Stan Development Team.*
